# Measuring grit, self-efficacy, curiosity, and intolerance of uncertainty in first-generation college and first-generation osteopathic medical students

**DOI:** 10.1186/s12909-023-04181-9

**Published:** 2023-03-28

**Authors:** DeWitt Jones, Monet McCalla, Elizabeth A. Beverly

**Affiliations:** 1 Department of Medicine, Ohio University Heritage College of Osteopathic Medicine, Athens, OH 45701 USA; 2 Department of Primary Care, Ohio University Heritage College of Osteopathic Medicine, Athens, OH 45701 USA; 3 The Diabetes Institute, Ohio University, Athens, OH 45701 USA

**Keywords:** Grit, Self-efficacy, Curiosity, Intolerance of uncertainty, Medical education

## Abstract

**Background:**

Medical school is a challenging time, with many medical students reporting symptoms of burnout, depression, anxiety, suicidal ideation, and psychological distress during pre-clinical and clinical years. First-generation college and first-generation medical students may be two groups of students at increased risk for the negative psychosocial effects of medical school. Importantly, grit, self-efficacy, and curiosity are protective factors against the negative psychosocial effects of medical school, whereas intolerance of uncertainty is a risk factor. Thus, research examining the associations among grit, self-efficacy, curiosity, and intolerance of uncertainty in first-generation college and first-generation medical students is needed.

**Methods:**

We conducted a cross-sectional, descriptive study to assess medical students’ grit, self-efficacy, curiosity, and intolerance of uncertainty. We conducted independent samples t-tests and regression analyses using SPSS statistical software version 28.0.

**Results:**

A total of 420 students participated in the study for a response rate of 51.5%. One-fifth of participants (21.2%, n = 89) identified as first-generation students, 38.6% (n = 162) participants reporting having a physician relative, and 16.2% (n = 68) reported having a physician parent. Grit, self-efficacy, and curiosity and exploration scores did not differ by first-generation college status, physician relative(s), or physician parent(s). However, total intolerance of uncertainty scores differed by physician relative(s) (t= -2.830, p = 0.005), but not by first-generation status, or physician parent(s). Further, subscale scores for prospective intolerance of uncertainty differed by physician relative(s) (t= -3.379, p = 0.001) and physician parent(s) (t= -2.077, p = 0.038), but not by first-generation college student status. In the hierarchical regression models, first-generation college student status and first-generation medical student status were not predictive of grit, self-efficacy, curiosity and exploration, or intolerance of uncertainty, although statistical trends were observed with students with physician relative(s) predicting lower intolerance of uncertainty scores (B= -2.171, t= -2138, p = 0.033) and lower prospective intolerance of uncertainty (B= -1.666, t= -2.689, p = 0.007).

**Conclusions:**

These findings suggest that first-generation college students did not differ by grit, self-efficacy, curiosity, or intolerance of uncertainty. Similarly, first-generation medical students did not differ by grit, self-efficacy, or curiosity; however, first-generation medical students showed statistical trends in higher total intolerance of uncertainty and higher prospective intolerance of uncertainty. Additional research needs to confirm these findings in first-generation medical students.

Medical school is a challenging time for students. Increasingly, medical students report symptoms of burnout, depression, anxiety, suicidal ideation, and psychological distress during pre-clinical and clinical years [1–4]. Prior research has identified specific factors that are protective against the negative psychosocial effects of medical school. A few of these factors include grit [5], self-efficacy [6, 7], and curiosity [8]. For example, grit serves as a protective factor against burnout [9] and self-efficacy lowers levels of psychological distress in medicals students [10]. Also, students with high levels of curiosity are able to leverage more personal and social resources in the face of stressful situations, which contributes to improved emotional well-being [11].

Conversely, intolerance of uncertainty is associated with increased burnout, depressive symptoms, and psychological distress in medical students [12–14]. Yet, the practice of medicine is intrinsically uncertain. This is pertinent considering medical students who struggle to cope with uncertainty experience increased psychological distress compared to students with a higher tolerance for uncertainty [12]. Interestingly, higher levels of grit, self-efficacy, and curiosity are associated with greater tolerance to uncertainty [15–17]. Further, higher levels of curiosity can resolve uncertainty by seeking new information to address gaps in knowledge [18, 19]. Thus, identifying students at increased risk for the negative mental and emotional effects of medical school is needed to provide additional support and resources to build grit, self-efficacy, curiosity, and tolerance for uncertainty.

First-generation college and first-generation medical students may be two groups at increased risk for the negative psychosocial effects of medical school. Findings from an Association of American Medical Colleges’ Pilot Survey found first-generation college students reported higher stress, fatigue, lower quality of life, and less social support than non-first-generation students [20]; the effects on first-generation medical school students is not known. For this reason, assessing levels of grit, self-efficacy, curiosity, and intolerance of uncertainty among first-generation college and first-generation medical students is important to identify potential differences and key areas for intervention. Therefore, we aimed to examine the relationship among grit, self-efficacy, curiosity, and intolerance of uncertainty in medical students. We examined uncertainty by first-generation college (i.e., defined by first person in nuclear family to earn a college degree) and first-generation medical students (i.e., define by students with and without physician parents and students with and without physician relatives). In addition, we examined associations among first-generation college and first-generation medical student status predicting grit, self-efficacy, curiosity and exploration, and intolerance of uncertainty, controlling for age, race, and year in medical school.

We hypothesized that first-generation medical student status would predict grit, self-efficacy, curiosity, and intolerance of uncertainty, but not first-generation college student status. We based this hypothesis on the large percentage of first-generation college students in the United States (i.e., 56%) along with recent efforts to target programming at institutions of higher education to reduce disparities between first-generation college students and non-first-generation college students [21]. Conversely, with minimal information available about this newly emerging group, termed first-generation medical students, we expected differences in these constructs.

## Methods

We utilized a descriptive, cross-sectional survey design to assess medical students’ grit, self-efficacy, curiosity, and intolerance of uncertainty. Specifically, we recruited medical students aged 18 years and older who were able to read and write in English.

### Ethics approval

Ethics approval for the study was obtained from the Ohio University Office of Research Compliance Institutional Review Board (approval number: 16-X-294). In complying with federal, state, and local laws and regulations for human subjects, we ensured our research met the requirements set forth in the regulations on public welfare in Part 46 of Title 45 of the Code of Federal Regulations (45 CFR 46); the principles set forth in “The Belmont Report,” and the Helsinki Declaration of 1975. Informed consent was obtained from all participants and all consented to participate in the study.

### Sample size

We conducted an a priori power analysis using G*Power 3 software [22], which determined a total sample size of 328 students (i.e., 66 first-generation college students and 262 non-first generation college students; 73 first-generation medical students and 109 non-first generation medical students) was estimated for 80% power at a 5% significance level (p < 0.05) to detect an effect size of d = 0.5.

### Participants

The electronic, anonymous survey was distributed to all medical students currently enrolled at the main campus (n = 569) and two distance campuses (A n = 146; B n = 101) of a large osteopathic medical school located in the United States. Osteopathic medical schools require the same rigorous training as allopathic medical schools. The main differences between osteopathic and allopathic medical schools include the following: (1) osteopathic medicine focuses on the whole body, and (2) osteopathic physicians have completed an additional 200 h of hands-on training with the musculoskeletal system known as osteopathic manipulative treatment. The email invitation was distributed by the study investigator (EAB) via school-maintained class lists. The survey opened on October 11, 2016 and a reminder email was sent on November 1, 2016. Participation in the study was completely voluntary.

### Measures

Participants completed a short demographic form (e.g., age, gender, race, ethnicity, year in medical school). No identifying information (i.e., name, email address, race/ethnicity) was collected in order to maintain anonymity. In addition, participants completed the following validated measures:

#### Grit scale

a 12-item scale measuring consistency of interests and perseverance of effort on a 5-point scale, ranging from 1= “Not at all like me” to 5= “Very much like me.” The Grit Scale has good internal consistency (α = 0.85) for the overall scale and for each factor (Consistency of Interests, = 0.84; Perseverance of Effort, α = 0.78) [23].

#### General self-efficacy scale

a 10-item scale measuring self-efficacy, or positive self-beliefs to cope with a difficult life demands, on a 4-point scale, ranging from 1=“Not at all true” to 4=“Exactly true.” The General Self-Efficacy Scale has good internal consistency ranging from α = 0.76 to α = 0.90 [24].

#### Curiosity and exploration inventory

a 7-item measure exploring recognition, pursuit, and integration of new and challenging experiences on a 7-point scale, ranging from 1= “Strongly disagree” to 7= “Strongly agree.” This inventory yields two factors: Exploration, or the pursuit of new experiences, and Absorption, or being engaged in activities. The scale demonstrates good convergent and discriminant validity as well as internal consistency (α = 0.80) [11].

#### Intolerance of uncertainty scale - short form

a 12-item scale measuring responses to uncertainty, ambiguous situations, and the future on a 5-point scale, ranging from 1 = “Not at all characteristic of me” to 5= “Entirely characteristic of me.” This scale yields two subscales: (1) Prospective Intolerance of Uncertainty Subscale, or fear in anticipation of future uncertainty, and (2) Inhibitory Intolerance of Uncertainty Subscale, inaction in the face of uncertainty. The scale demonstrates good convergent and discriminant validity as well as internal consistency (α = 0.85) [25]. Higher scores indicate higher intolerance of uncertainty.

### Data collection

Participants completed the survey online without identifying information via the online questionnaire service Qualtrics (Provo, UT: Qualtrics). Consent occurred online. As in previous studies conducted by the research team [26–30], participants clicked a radio button indicating “Yes, I consent to participate in this study. I may withdraw my participation at any time.“ Conversely, if participants declined to participate, they clicked a radio button indicating “I decline to participate.“ Both the online welcome screen for the survey and the informed consent document specified the voluntary nature of participation. To avoid coercion, no researchers were present when individuals elected to participate or decline, which may have generated less pressure than in a face-to-face consent process. All participants were instructed to contact the research investigators by email or telephone if they had any questions. Completion of the entire survey took approximately 30 min. Participants received a $15.00 gift card as human subject compensation. To receive the gift card, participants clicked on a new Qualtrics link where they could provide an email address to receive a gift card. This step was necessary to that their responses were not linked to their email address.

### Data analysis

Basic sociodemographic characteristics of participants were assessed using descriptive statistics. Frequencies of individual question responses were also calculated. Next, we calculated survey scores for the Total Grit Scale, Grit Subscale: Consistency of Interests, Grit Subscale Perseverance of Effort, General Self-Efficacy Scale, Curiosity and Exploration Inventory, Exploration Subscale, and Absorption Subscale, Intolerance of Uncertainty, Prospective Intolerance of Uncertainty Subscale, and Inhibitory Intolerance of Uncertainty Subscale. For missing data, we specified pairwise deletion to maximize all data available on an analysis by analysis basis. Next, we conducted bivariate (Pearson) correlations to determine associations among the survey scores and age. Correlations were evaluated by the strength of the correlation coefficient (negligible = 0.0-0.3, low-0.3-0.5, moderate = 0.5–0.7, high = 0.7–0.9, very high = 0.9-1.0) [31]. Independent samples t-tests were used to examine mean differences in grit, self-efficacy, curiosity and exploration, and intolerance of uncertainty by sociodemographic categories and by first-generation college status (i.e., first person in nuclear family to earn a college degree), and first-generation medical student status (i.e., students with and without parent physician(s) and students with and without physician relative(s). Finally, we conducted hierarchical linear regression models to examine associations among first-generation college and first-generation medical student status predicting grit, self-efficacy, curiosity and exploration, and intolerance of uncertainty, controlling for age, gender, race, and year in medical school. For gender, race, ethnicity, and year in medical school we created dummy variables for each category, with the category = 1 and all other data = 0 (e.g., Year 1 = 1, All other years = 0). Statistically significant variables from the bivariate correlations and independent t-tests were included in the regression models. Statistical significance was defined as *p* < 0.05 unless a Bonferroni correction was used. All analyses were conducted with SPSS statistical software version 28.0 (Chicago, IL: SPSS Inc.).

## Results

Of the 816 osteopathic medical students enrolled at the three campuses, 420 students participated in the study for a response rate of 51.5%. The mean age of the participants was 25.4 ± 3.2 years, 55.5% (n = 233) identified as women, 44.3% (n = 186) identified as men, and 0.2% (n = 1) reported an identity not listed. The self-reported racial distribution of the sample included 0.5% (n = 2) American Indian/Alaskan Native, 8.3% Asian (n = 35), 7.1% (n = 30) Black/African American, 0.5% (n = 2) Native Hawaiian/Pacific Islander, 4.8% (n = 20) Other, and 78.3% (n = 329) White; and 4.3% (n = 18) identified as Hispanic/Latino. The distribution by year in medical school was 37.4% (n = 157) for first-year, 31.2% (n = 131), 18.8% (n = 79), and 12.6% (n = 53). Note, the third- and fourth-year medical student classes did not have students on the three campuses, thus their class sizes were much smaller. Finally, one-fifth of participants (21.2%, n = 89) identified as first-generation students, 38.6% (n = 162) participants reporting having a physician relative, and 16.2% (n = 68) reported having a physician parent. Additional demographic data are presented in Table 1.

**Table 1:** Participant Demographics (n = 420)

Variable	Participants n (%)
Age (years)	25.4 ± 3.2
Gender	
Female	233 (55.5)
Male	186 (44.3)
An identity not listed	1 (0.2)
Prefer not to answer	0 (0)
Race	
American Indian/Alaskan Native	2 (0.5)
Asian	35 (8.3)
Black/African American	30 (7.1)
Native Hawaiian/Pacific Islander	2 (0.5)
Other	20 (4.8)
White/Caucasian	329 (78.3)
Ethnicity	
Hispanic/Latino	18 (4.3)
Year in medical school	
Year 1	157 (37.4)
Year 2	131 (31.2)
Year 3	79 (18.8)
Year 4	53 (12.6)
First generation student	
Yes	89 (21.2)
No	330 (78.6)
Physician parent(s)	
Yes	68 (16.2)
No	352 (83.8)
Physician relative(s)	
Yes	162 (38.6)
No	258 (61.4)

### Correlational findings

Grit had negligible to low correlations with self-efficacy (r = 0.323; see Table 2), curiosity and exploration (r = 0.131), absorption (r = 0.126), intolerance of uncertainty (r= -0.221), prospective intolerance of uncertainty (r= -0.117), and inhibitory intolerance of uncertainty (r= -0.311). Consistency was correlated with self-efficacy (r = 0.212), intolerance of uncertainty (r= -0.223), prospective intolerance of uncertainty (r= -0.140), inhibitory intolerance of uncertainty (r= -0.228), and age (r= -0.120). Similarly, perseverance was correlated with self-efficacy (r = 0.324), curiosity and exploration (r = 0.167), exploration (r = 0.161), absorption (r = 0.116), intolerance of uncertainty (r= -0.124). inhibitory intolerance of uncertainty (r= -0.208), and age (r = 0.102). Similarly, self-efficacy had negligible to low correlations with curiosity and exploration (r = 0.359), exploration (r = 0.319), absorption (r = 0.273), intolerance of uncertainty (r= -0.263), prospective intolerance of uncertainty (r= -0.161), and inhibitory intolerance of uncertainty (r= -0.343). Curiosity and exploration was correlated with inhibitory intolerance of uncertainty (r= -0.104) and age (r= -0.125). Interestingly, exploration was correlated with inhibitory intolerance of uncertainty (r= -0.131), and absorption was correlated with prospective intolerance of uncertainty (r = 0.151) and age (r= -0.141). Finally, intolerance of uncertainty and prospective intolerance of uncertainty were correlated with age (r= -0.115, r= -0.114), respectively.

**Table 2:** Intercorrelations among Psychological Characteristics and Age (n = 392)

	Consistency	Perseverance	Self-Efficacy	CEI	Exploration	Absorption	IUS	Prospective IUS	Inhibitory IUS	Age
Grit	0.856***	0.750***	0.323***	0.131**	0.094	0.126*	-0.221***	-0.117*	-0.311***	-0.028
Consistency		0.300***	0.212***	0.058	0.009	0.091	-0.223***	-0.140**	-0.286***	-0.120*
Perseverance			0.324***	0.167***	0.161**	0.116**	-0.124*	-0.038	-0.208***	0.102*
Self-Efficacy				0.359***	0.319***	0.273***	-0.263***	-0.161**	-0.343***	-0.040
CEI					0.842***	0.821***	-0.001	0.082	-0.104*	-0.125*
Exploration						0.384***	-0.071	-0.012	-0.131**	-0.095
Absorption							0.073	0.151**	-0.038	-0.114*
IUS								0.936***	0.899***	-0.115*
Prospective									0.689***	-0.114*
Inhibitory										-0.096

*p < 0.05, **p < 0.01,***p < 0.001; CEI = Curiosity and Exploration Inventory; IUS = Intolerance of Uncertainty Scale

### Grit

The total mean for grit was 3.7 ± 0.5, and the subscales scores for consistency and perseverance were 3.4 ± 0.7 and 4.1 ± 0.5, respectively (see Table 3). Grit scores did not differ by first-generation college status (t= -0.283, p = 0.778), physician relative(s) (t = 0.057, p = 0.955), or physician parent(s) (t= -0.844, p = 0.399).

**Table 3:** Mean Differences in Grit, Self-Efficacy, Curiosity and Exploration, and Intolerance of Uncertainty Scores by Sociodemographic Variables and First-Generation Statuses (n = 392)

	Grit M ± SD	Consistency M ± SD	Perseverance M ± SD	Self-Efficacy M ± SD	Curiosity & Exploration M ± SD	Exploration M ± SD	Absorption M ± SD	Intolerance of Uncertainty M ± SD	Prospective M ± SD	Inhibitory M ± SD
**Total**										
All participants	3.7 ± 0.5	3.4 ± 0.7	4.1 ± 0.5	32.3 ± 4.7	34.2 ± 5.5	20.6 ± 3.4	13.5 ± 3.2	30.0 ± 8.5	19.6 ± 5.2	10.4 ± 4.1
**Gender** ^**‡**^										
Men	3.7 ± 0.5*	3.3 ± 0.7*	4.0 ± 0.6	33.1 ± 4.8**	34.2 ± 5.3	20.6 ± 3.3	13.6 ± 3.1	29.3 ± 8.2	19.0 ± 5.0	10.3 ± 3.9
Women	3.8 ± 0.5	3.4 ± 0.7	4.1 ± 0.5	31.8 ± 4.2	34.3 ± 5.5	20.7 ± 3.4	13.5 ± 3.2	30.5 ± 8.8	20.0 ± 5.3	10.5 ± 4.3
**Race** ^**‡**^										
American Indian/Alaskan Native	3.6 ± 0.8	2.7 ± 2.4	4.5 ± 0.7	23.5 ± 19.1***	26.5 ± 14.8*	16.5 ± 6.4	10.0 ± 8.5	36.5 ± 10.6	22.0 ± 1.4	14.5 ± 9.2
Asian	3.4 ± 0.6***	2.9 ± 0.8***	3.8 ± 0.7**	29.5 ± 5.8***	36.0 ± 4.9*	21.7 ± 3.2	14.3 ± 2.8	37.1 ± 8.5***	22.9 ± 5.1***	14.2 ± 4.3***
Black/African American	4.0 ± 0.5**	3.8 ± 0.7**	4.2 ± 0.5	33.3 ± 4.3	31.8 ± 6.4**	18.8 ± 3.9**	13.0 ± 3.3	28.4 ± 9.0	19.3 ± 6.3	9.1 ± 3.5
Native Hawaiian/Pacific Islander	3.7 ± 0.5	3.3 ± 0.4	4.1 ± 0.6	34.0 ± 2.8	38.5 ± 3.5	23.0 ± 5.7	15.5 ± 2.1	32.0 ± 4.2	22.0 ± 1.4	10.0 ± 2.8
White	3.8 ± 0.5*	3.4 ± 0.6*	4.1 ± 0.5	32.6 ± 4.1*	34.3 ± 5.3	20.8 ± 3.3	13.5 ± 3.2	29.2 ± 8.1***	19.1 ± 5.0**	10.0 ± 3.9***
Other	3.6 ± 0.4	3.1 ± 0.6	4.0 ± 0.5	31.7 ± 7.8	31.8 ± 5.3	18.9 ± 3.4*	12.9 ± 3.4	31.6 ± 10.3	20.3 ± 5.5	11.3 ± 5.3
**Ethnicity** ^**‡**^										
Hispanic/Latino	3.7 ± 0.5	2.9 ± 1.0*	4.4 ± 0.5*	29.5 ± 8.3*	31.2 ± 7.7	19.1 ± 4.6	12.1 ± 4.0	31.3 ± 11.3	20.3 ± 6.6	10.9 ± 5.5
**Year in school** ^**‡**^										
Year 1	3.8 ± 0.5*	3.4 ± 0.7	4.2 ± 0.5**	32.3 ± 5.2	34.4 ± 5.7	20.7 ± 3.7	13.7 ± 3.2	29.6 ± 8.8	19.3 ± 5.4	10.2 ± 4.2
Year 2	3.7 ± 0.5	3.4 ± 0.7	4.0 ± 0.6	32.6 ± 4.3	34.5 ± 5.4	20.7 ± 3.4	13.8 ± 3.1	30.0 ± 8.2	19.7 ± 4.9	10.3 ± 4.0
Year 3	3.7 ± 0.5	3.4 ± 0.6	4.1 ± 0.5	31.9 ± 4.0	34.2 ± 5.1	20.5 ± 3.1	13.6 ± 3.1	31.2 ± 9.1	20.2 ± 5.5	10.9 ± 4.3
Year 4	3.6 ± 0.5*	3.2 ± 0.8*	4.0 ± 0.5	31.9 ± 4.9	32.7 ± 5.6*	20.3 ± 3.0	12.4 ± 3.5*	29.2 ± 7.8	18.9 ± 4.7	10.3 ± 4.1
**First-generation college students** ^**‡**^										
Yes	3.7 ± 0.5	3.4 ± 0.7	4.1 ± 0.6	32.2 ± 5.1	34.3 ± 6.1	20.5 ± 3.6	13.7 ± 3.4	30.6 ± 8.8	19.9 ± 5.2	10.7 ± 4.3
No	3.7 ± 0.5	3.4 ± 0.7	4.1 ± 0.5	32.3 ± 4.6	34.2 ± 5.3	20.7 ± 3.3	13.5 ± 3.2	29.8 ± 8.5	19.5 ± 5.1	10.3 ± 4.1
**Physician parent(s)** ^**‡**^										
Yes	3.7 ± 0.5	3.3 ± 0.8	4.1 ± 0.5	32.7 ± 4.9	34.0 ± 5.9	20.5 ± 3.6	13.5 ± 3.6	28.1 ± 8.9	18.3 ± 5.4*	9.8 ± 4.4
No	3.7 ± 0.5	3.4 ± 0.7	4.1 ± 0.5	32.2 ± 4.6	34.2 ± 5.4	20.7 ± 3.3	13.6 ± 3.1	30.3 ± 8.4	19.8 ± 5.1	10.5 ± 4.1
**Physician relative(s)** ^**‡**^										
Yes	3.7 ± 0.5	3.4 ± 0.7	4.1 ± 0.6	32.5 ± 4.7	34.4 ± 5.8	20.9 ± 3.7	13.5 ± 3.3	28.5 ± 8.9**	18.5 ± 5.4***	10.0 ± 4.3
No	3.7 ± 0.5	3.4 ± 0.6	4.1 ± 0.5	32.2 ± 4.7	34.0 ± 5.3	20.5 ± 3.2	13.5 ± 3.2	31.0 ± 8.2	20.3 ± 4.9	10.7 ± 4.0

*p < 0.05,**p < 0.01,***p < 0.001; M = Mean; SD = Standard Deviation; ^**‡**^Variables recoded as dummy variables with each variable category = 1 and all other data coded = 0

The overall regression model was significant (F(11,376) = 4.44, p < 0.001; see Table 4). In Step 1 (Bonferroni correction was p < 0.00833), Black/African American race (B = 0.399, t = 2.721, p = 0.007) was independently associated with total grit scores. In step 2 (Bonferroni correction was p < 0.00625), gender (B= -0.128, t= -2.623, p = 0.004) was the only statistically significant predictor of grit. Finally, in step 3 (Bonferroni correction was p < 0.00454), gender remained the only significant predictor of grit.

**Table 4:** Summary of Hierarchical Regression Analyses Examining the Associations among Sociodemographic Characteristics and First-Generation Statuses with Grit, Self-Efficacy, Curiosity and Exploration, and Intolerance of Uncertainty (n = 387)

	Dependent Variable											
**Independent Variables** ^‡^	**Step 1** ^**a**^				**Step 2** ^**b**^				**Step 3** ^**c**^			
***Model 1: Grit***	**B**	**SE**	**t**	**p**	**B**	**SE**	**t**	**p**	**B**	**SE**	**t**	**p**
Age	-0.008	0.008	-0.927	0.354	0.002	0.009	0.257	0.797	0.002	0.009	0.179	0.858
Gender	-0.128	0.049	-2.623	0.009	-0.138	0.048	-2.860	0.004	-0.141	0.048	-2.919	0.004
Asian	-0.252	0.135	-1.869	0.062	-0.251	0.134	-1.874	0.062	-0.264	0.134	-1.964	0.050
Black/African American	0.399	0.147	2.721	0.007	0.384	0.146	2.633	0.009	0.385	0.147	2.622	0.009
White	0.151	0.110	1.375	0.170	0.156	0.110	1.421	0.156	0.150	0.110	1.364	0.173
Hispanic/Latino	0.067	0.130	0.516	0.606	0.030	0.130	0.228	0.819	0.042	0.130	0.325	0.746
Year 1					0.122	0.053	2.304	0.022	0.123	0.053	2.309	0.021
Year 4					-0.128	0.078	-1.652	0.099	-0.121	0.078	-1.550	0.122
First-generation college									-0.044	0.061	-0.714	0.476
Physicians in family									0.033	0.058	0.566	0.572
Physician parents									-0.103	0.077	-1.351	0.178
***Model 1a: Consistency***	**B**	**SE**	**t**	**p**	**B**	**SE**	**t**	**p**	**B**	**SE**	**t**	**p**
Age	-0.024	0.011	-2.121	0.035	-0.016	0.012	-1.313	0.190	-0.018	0.012	-1.440	0.151
Gender	-0.158	0.066	-2.380	0.018	-0.165	0.066	-2.488	0.013	-0.170	0.066	-2.571	0.011
Asian	-0.332	0.183	-1.809	0.071	-0.344	0.184	-1.869	0.062	-0.372	0.184	-2.021	0.044
Black/African American	0.563	0.200	2.818	0.005	0.537	0.201	2.676	0.008	0.526	0.201	2.616	0.009
White	0.176	0.150	1.171	0.242	0.167	0.151	1.105	0.270	0.158	0.151	1.048	0.295
Hispanic/Latino	-0.157	0.178	-0.887	0.376	-0.194	0.178	-1.089	0.277	-0.173	0.178	-0.969	0.333
Year 1					0.059	0.073	0.816	0.415	0.056	0.073	0.776	0.438
Year 4					-0.163	0.107	-1.528	0.127	-0.153	0.107	-1.428	0.154
First-generation college									-0.033	0.084	-0.389	0.698
Physicians in family									0.088	0.080	1.108	0.269
Physician parents									-0.249	0.105	-2.379	0.018
***Model 1b: Perseverance***	**B**	**SE**	**t**	**p**	**B**	**SE**	**t**	**p**	**B**	**SE**	**t**	**p**
Age	0.009	0.009	0.938	0.349	0.021	0.010	2.106	0.036	0.021	0.010	2.110	0.036
Gender	-0.098	0.054	-1.811	0.071	-0.111	0.053	-2.088	0.037	-0.112	0.053	-2.104	0.036
Asian	-0.171	0.149	-1.151	0.250	-0.158	0.147	-1.070	0.285	-0.156	0.148	-1.055	0.292
Black/African American	0.235	0.162	1.448	0.148	0.232	0.161	1.443	0.150	0.244	0.162	1.509	0.132
White	0.127	0.122	1.044	0.297	0.145	0.121	1.202	0.230	0.142	0.121	1.173	0.241
Hispanic/Latino	0.292	00.144	2.026	0.043	0.253	0.143	1.774	0.077	0.257	0.144	1.791	0.074
Year 1					0.184	0.058	3.165	0.002	0.189	0.059	3.225	0.001
Year 4					-0.094	0.086	-1.094	0.275	-0.090	0.086	-1.040	0.299
First-generation college									-0.055	0.067	-0.813	0.417
Physicians in family									-0.022	0.064	-0.348	0.728
Physician parents									0.042	0.084	0.502	0.616
***Model 2: Self-Efficacy***	**B**	**SE**	**t**	**p**	**B**	**SE**	**t**	**p**	**B**	**SE**	**t**	**p**
Age	0.029	0.079	0.367	0.714	0.033	0.085	0.387	0.699	0.036	0.085	0.424	0.672
Gender	1.177	0.459	2.563	0.011	1.173	0.462	2.541	0.011	1.190	0.464	2.566	0.011
Asian	-2.581	1.250	-2.064	0.040	-2.581	1.260	-2.048	0.041	-2.523	1.268	-1.990	0.047
Black/African American	1.002	1.378	0.727	0.467	0.998	1.390	0.718	0.473	0.969	1.401	0.692	0.489
White	0.227	1.014	0.224	0.823	0.228	1.025	0.223	0.824	0.263	1.029	0.255	0.799
Hispanic/Latino	-1.397	1.226	-1.140	0.255	-1.412	1.236	-1.142	0.254	-1.480	1.245	-1.189	0.235
Year 1					0.040	0.507	0.079	0.937	0.025	0.510	0.049	0.961
Year 4					-0.058	0.748	-0.078	0.938	-0.096	0.753	-0.127	0.899
First-generation college									0.360	0.589	0.611	0.541
Physicians in family									-0.030	0.557	-0.054	0.957
Physician parents									0.345	0.733	0.470	0.639
***Model 3: CEI***	**B**	**SE**	**t**	**p**	**B**	**SE**	**t**	**p**	**B**	**SE**	**t**	**p**
Age	0-0.084	0.095	-0.889	0.375	-0.052	0.101	-0.515	0.607	-0.053	0.102	-0.525	0.600
Gender	-0.128	0.551	-0.232	0.816	-0.146	0.553	-0.263	0.792	-0.139	0.555	-0.250	0.803
Asian	2.781	1.523	1.826	0.069	2.661	1.532	1.737	0.083	2.662	1.539	1.729	0.085
Black/African American	-1.253	1.659	-0.755	0.451	-1.438	1.671	-0.860	0.390	-1.558	1.681	-0.926	0.355
White	1.126	1.246	0.904	0.367	1.014	1.256	0.808	0.420	1.070	1.260	0.849	0.397
Hispanic/Latino	-1.824	1.473	-1.238	0.217	-2.000	1.483	-1.348	0.178	-2.016	1.491	-1.352	0.177
Year 1					0.003	0.605	0.004	0.997	-0.057	0.608	-0.094	0.925
Year 4					-0.981	0.896	-1.095	0.274	-1.000	0.901	-1.110	0.268
First-generation college									0.672	0.703	0.955	0.340
Physicians in family									0.567	0.666	0.852	0.395
Physician parents									-0.687	0.877	-0.784	0.434
***Model 3a: Exploration***	**B**	**SE**	**t**	**p**	**B**	**SE**	**t**	**p**	**B**	**SE**	**t**	**p**
Age	-0.019	0.059	-0.316	0.752	-0.014	0.063	-0.218	0.827	-0.015	0.063	-0.238	0.812
Gender	-0.206	0.342	-0.601	0.548	-0.213	0.344	-0.619	0.536	-0.224	0.345	-0.651	0.516
Asian	2.068	0.946	2.187	0.029	2.091	0.953	2.195	0.029	2.084	0.957	2.178	0.030
Black/African American	-0.843	1.030	-0.819	0.413	-0.825	1.039	-0.794	0.428	-0.837	1.045	-0.801	0.424
White	1.163	0.774	1.503	0.134	1.187	0.781	1.520	0.129	1.217	0.783	1.554	0.121
Hispanic/Latino	-0.966	0.915	-1.055	0.292	-0.973	0.923	-1.055	0.292	-0.921	0.927	-0.994	0.321
Year 1					0.130	0.376	0.345	0.731	0.102	0.378	0.271	0.786
Year 4					0.041	0.558	0.073	0.942	0.067	0.560	0.119	0.905
First-generation college									0.185	0.437	0.424	0.672
Physicians in family									0.594	0.414	1.433	0.153
Physician parents									-0.637	0.545	-1.170	0.243
***Model 3b: Absorption***	**B**	**SE**	**t**	**p**	**B**	**SE**	**t**	**p**	**B**	**SE**	**t**	**p**
Age	-0.066	0.056	-1.170	0.243	-0.038	0.060	-0.642	0.521	-0.038	0.060	-0.639	0.523
Gender	0.078	0.326	0.238	0.812	0.067	0.326	0.206	0.837	0.086	0.328	0.262	0.794
Asian	0.713	0.901	0.791	0.430	0.570	0.903	0.631	0.529	0.578	0.908	0.636	0.525
Black/African American	-0.409	0.982	-0.417	0.677	-0.613	0.985	-0.622	0.535	-0.721	0.992	-0.726	0.468
White	-0.037	0.737	-0.050	0.960	-0.173	0.741	-0.233	0.816	-0.148	0.744	-0.199	0.842
Hispanic/Latino	-0.858	0.872	-0.984	0.326	-1.026	0.875	-1.173	0.242	-1.094	0.880	-1.244	0.214
Year 1					-0.127	0.357	-0.356	0.722	-0.160	0.359	-0.445	0.656
Year 4					-1.022	0.529	-1.933	0.054	-1.067	0.532	-2.006	0.046
First-generation college									0.487	0.415	1.173	0.242
Physicians in family									-0.026	0.393	-0.067	0.947
Physician parents									-0.050	0.517	-0.097	0.923
***Model 4: IUS***	**B**	**SE**	**t**	**p**	**B**	**SE**	**t**	**p**	**B**	**SE**	**t**	**p**
Age	-0.394	0.146	-2.698	0.007	-0.453	0.156	-2.896	0.004	-0.470	0.155	-3.025	0.003
Gender	-0.899	0.850	-1.058	0.291	-0.808	0.851	-0.950	0.343	-0.761	0.846	-0.900	0.369
Asian	4.727	2.345	2.016	0.045	4.448	2.354	1.889	0.060	4.036	2.344	1.722	0.086
Black/African American	-2.756	2.578	-1.069	0.286	-3.034	2.592	-1.171	0.243	-3.540	2.583	-1.371	0.171
White	-2.805	1.918	-1.462	0.145	-3.106	1.931	-1.609	0.109	-3.349	1.920	-1.745	0.082
Hispanic/Latino	0.284	2.269	0.125	0.900	0.375	2.280	0.164	0.869	0.165	2.272	0.073	0.942
Year 1					-1.566	0.933	-1.677	0.094	-1.570	0.929	-1.690	0.092
Year 4					-0.522	1.378	-0.379	0.705	-0.674	1.373	-0.491	0.624
First-generation college									0.184	1.073	0.171	0.864
Physicians in family									-2.171	1.015	-2.138	0.033
Physician parents									-0.669	1.335	-0.501	0.617
***Model 4a: Prospective IUS***	**B**	**SE**	**t**	**p**	**B**	**SE**	**t**	**p**	**B**	**SE**	**t**	**p**
Age	-0.232	0.089	-2.595	0.010	-0.258	0.096	-2.698	0.007	-0.271	0.095	-2.855	0.005
Gender	-0.837	0.520	-1.608	0.109	-0.790	0.521	-1.515	0.131	-0.755	0.516	-1.463	0.144
Asian	1.910	1.436	1.330	0.184	1.734	1.443	1.201	0.230	1.443	1.430	1.009	0.314
Black/African American	-0.968	1.579	-0.613	0.540	-1.155	1.589	-0.727	0.468	-1.506	1.575	-0.956	0.340
White	-1.535	1.175	-1.307	0.192	-1.722	1.183	-1.456	0.146	-1.903	1.171	-1.625	0.105
Hispanic/Latino	0.590	1.390	0.424	0.672	0.607	1.397	0.434	0.664	0.450	1.386	0.325	0.746
Year 1					-0.864	0.572	-1.511	0.132	-0.860	0.567	-1.517	0.130
Year 4					-0.472	0.845	-0.559	0.577	-0.583	0.837	-0.697	0.486
First-generation college									0.070	0.655	0.107	0.915
Physicians in family									-1.666	0.619	-2.689	0.007
Physician parents									-0.333	0.815	-0.409	0.682
***Model 4b: Inhibitory IUS***	**B**	**SE**	**t**	**p**	**B**	**SE**	**t**	**p**	**B**	**SE**	**t**	**p**
Age	-0.162	0.070	-2.312	0.021	-0.194	0.075	-2.590	0.010	-0.199	0.075	-2.656	0.008
Gender	-0.062	0.407	-0.152	0.879	-0.018	0.408	-0.044	0.965	-0.006	0.409	-0.015	0.988
Asian	2.817	1.124	2.506	0.013	2.715	1.129	2.405	0.017	2.593	1.133	2.289	0.023
Black/African American	-1.788	1.236	-1.446	0.149	-1.880	1.243	-1.512	0.131	-2.034	1.248	-1.629	0.104
White	-1.269	0.920	-1.380	0.168	-1.383	0.926	-1.494	0.136	-1.447	0.928	-1.559	0.120
Hispanic/Latino	-0.306	1.088	-0.281	0.779	0-0.232	1.094	-0.212	0.832	-0.285	1.098	0-0.260	0.795
Year 1					-0.701	0.448	-1.567	0.118	-0.710	0.449	-1.581	0.115
Year 4					-0.050	0.661	-0.075	0.940	-0.090	0.663	-0.136	0.892
First-generation college									0.114	0.519	0.219	0.826
Physicians in family									-0.505	0.491	-1.030	0.304
Physician parents									-0.335	0.645	-0.519	0.604

CEI = Curiosity and Exploration Inventory; IUS = Intolerance of Uncertainty Scale; ^**‡**^Categorical variables recoded as dummy variables with each variable category = 1 and all other data coded = 0; Gender: men = 1, women = 0

^a^ The critical p-value for step 1 of the regression analyses was corrected to 0.00833 using the Bonferroni correction to account for six independent variables

^b^ The critical p-value for step 2 of the regression analyses was corrected to 0.00625 using the Bonferroni correction to account for eight independent variables

^c^ The critical p-value for step 3 of the regression analyses was corrected to 0.00454 using the Bonferroni correction to account for eleven independent variables

For the grit subscales, no factors were predictive of consistency in step 3 of the model. However, self-identifying as Hispanic/Latino was a significant predictor of higher perseverance scores in step 3 of the model (B = 0.189, t = 3.225, p = 0.001).

### General self-efficacy

The mean for general self-efficacy was 32.3 ± 4.7. General self-efficacy scores did not differ by first-generation college status (t= -0.117, p = 0.907), physician relative(s) (t = 0.581, p = 0.561), or physician parent(s) (t = 0.744, p = 0.457).

The overall regression model was significant (F(11,375) = 2.167, p = 0.016; see Table 4); however, with the Bonferroni corrections none of the first-generation status variables were independent predictors of self-efficacy in the regression models. Further, no other sociodemographic variables predicted self-efficacy.

### Curiosity and exploration

The mean for the curiosity and exploration inventory was 34.2 ± 0.5. The subscale score for exploration was 20.6 ± 3.4, and the subscale score for absorption was 13.5 ± 3.2. Total curiosity and exploration scores did not differ by first-generation college status (t = 0.185, p = 0.853), physician relative(s) (t = 0.656, p = 0.512), or physician parent(s) (t= -0.289, p = 0.772). Similarly, exploration and absorption scores did not differ by first-generation college status (t= -0.301, p = 0.763; t = 0.636, p = 0.525), physician relative(s) (t = 1.069, p = 0.286; t= -0.007, p = 0.995), or physician parent(s) (t= -0.297, p = 0.767; t= -0.181, p = 0.856), respectively.

The curiosity and exploration regression model was not statistically significant (F(11,375) = 1.454, p = 0.147; see Table 4). In steps 1, 2 and 3 of the models, no factors independently predicted total curiosity and exploration scores. Similarly, the final models for the exploration subscale (F(11,375) = 1.664, p = 0.080) and absorption subscale (F(11,375) = 0.967, p = 0.477) were not statistically significant and no factors predicted exploration or absorption scores.

### Intolerance of uncertainty

The mean for intolerance of uncertainty was 30.0 ± 8.5. Subscale scores for prospective intolerance of uncertainty were 19.6 ± 5.2 and scores for inhibitory intolerance of uncertainty were 10.4 ± 4.1. Total intolerance of uncertainty scores differed by physician relative(s) (t= -2.830, p = 0.005), but not by first-generation college status (t = 0.749, p = 0.454) or physician parent(s) status (t= -1.934, p = 0.054). Further, prospective intolerance of uncertainty scores differed by physician relative(s) (t= -3.379, p < 0.001) and physician parent(s) (t= -2.077, p = 0.038), but not by first-generation college status (t = 0.746, p = 0.456)). Inhibitory intolerance of uncertainty scores did not differ by any of the first-generation statuses. Thus, means scores for total intolerance of uncertainty scores and prospective intolerance of uncertainty scores differed by first-generation medical student status, but not inhibitory intolerance of uncertainty scores.

The overall regression model was statistically significant (F(11,374) = 4.509, p < 0.001; see Table 4). In step 1 (Bonferroni correction was p < 0.00833), only age (B= -0.394, t= -2.698, p = 0.007) was an independent predictor of intolerance of uncertainty. In step 2 (Bonferroni correction was p < 0.00625), age (B= -0.453, t= -2.896, p = 0.004) remained a significant predictor of intolerance of uncertainty. Finally, in step 3 (Bonferroni correction was p < 0.00454), a statistical trend was observed with students with physician relative(s) predicting lower intolerance of uncertainty scores (B= -2.171, t= -2138, p = 0.033); age remained the only significant predictor of intolerance of uncertainty in the final model.

For the final model predicting prospective intolerance of uncertainty (F(11,374) = 3.806, p < 0.001; see Table 4), both age (B= -0.271, t= -2.855, p = 0.005) and physician relative(s) status (B= -0.1.666, t= -2.689, p = 0.007) demonstrated statistical trends. No factors predicted inhibitory intolerance of uncertainty in the final regression model.

## Discussion

This descriptive, cross-sectional study measured grit, self-efficacy, curiosity, and intolerance of uncertainty in medical students at a three-campus medical school in the United States. All four constructs are known factors associated with psychosocial well-being [5–8] as well as academic success, perseverance, and productivity [11, 23–25, 32–35]. Grit, self-efficacy, and curiosity and exploration scores did not differ by first-generation college status, physician relative(s), or physician parent(s). However, we observed mean differences between students with physician relative(s) and total intolerance of uncertainty. Further, we observed differences in prospective intolerance of uncertainty by students with physician relative(s) and physician parent(s). These findings indicate that first-generation medical students reported higher total intolerance of uncertainty and higher prospective intolerance of uncertainty, or fear in anticipation of future uncertainty. Next, we examined associations among first-generation statuses predicting grit, self-efficacy, curiosity and exploration, and intolerance of uncertainty, controlling for sociodemographic variables. In the regression models, first-generation college student status and first-generation medical student status did not predict grit, self-efficacy, curiosity and exploration, or intolerance of uncertainty, although statistical trends were observed with students with physician relative(s) predicting lower intolerance of uncertainty scores and lower prospective intolerance of uncertainty. Overall, these findings suggest that higher education’s efforts to level the playing field for first-generation college students are working, and first-generation college students may not be at a disadvantage with respect to these four psychological constructs that contribute to psychosocial well-being and academic success. More research on first-generation medical students is needed before definitive associations with intolerance of uncertainty can be determined.

As hypothesized, no differences were observed by first-generation college and non-first-generation college participants in grit, self-efficacy, curiosity, or intolerance of uncertainty. This finding may be attributed to higher education’s initiatives designed to address first-generation college students’ financial barriers, psychological stressors, lack of social networks, imposter syndrome, and college readiness [36–38]. While this finding cannot be generalized to all medical students, it shows that the participating first-generation college students in this study were not at a disadvantage compared to non-first-generation college students in factors associated with academic success and psychological well-being. This finding may indicate a “turning of the tide” in first-generation college student research. Importantly, the first-generation college students who matriculate to medical school likely represent a distinctive group of first-generation college students who possess more grit, self-efficacy, and curiosity and less intolerance of uncertainty to navigate the rigorous pre-medical programming and medical admission tests. While these findings suggest that certain historical disadvantages between first-generation college students and non-first-generation students are diminishing, additional research with a larger, more diverse population of students is needed before any conclusions can be determined. Further, this unique group should be examined to determine what and how they overcame psychological, academic, financial, and social barriers to enter medical school.

We also examined a newly emerging group of first-generation students that we termed first-generation medical students. To date minimal research has examined differences between medical students with and without physicians in the family. Contrary to our hypothesis, we found no differences in grit, self-efficacy, or curiosity between first-generation medical students and non-first-generation medical students. This may be explained by the associations between those constructs and strong academic performance [11, 23–25, 32–35], which is a prerequisite for admission to medical school [39, 40]. The main difference we observed was in total intolerance of uncertainty and prospective intolerance of uncertainty. Students with physicians in the family reported higher mean total intolerance of uncertainty. Both students with physician parents and with physicians in the family reported higher prospective intolerance of uncertainty. However, in our regression model, neither students with physician parents nor students with physicians in the family predicted intolerance of uncertainty and its subscales, although we observed statistical trends with physicians in the family and intolerance of uncertainty and prospective intolerance of uncertainty.

Our findings showed that both first-generation college student status and first-generation medical student status were not associated with grit, self-efficacy, or curiosity and exploration. In contrast, first-generation medical student status was associated with higher intolerance of uncertainty and higher prospective intolerance of uncertainty, and a statistical trend persisted in the final regression model. Potential explanations for this difference include first-generation medical students may feel uncertain or anxious about their future if they lack a safety net for unexpected difficulties that arise in medical school. This may add a layer of stress and pressure not felt by non-first-generation medical students. Another contributor of increased uncertainty about the future may be a lack of networking in the medical field [41]. Medical school is anxiety-provoking on its own; however, navigating medical school admissions, board examinations, audition rotations, and residency applications without a close physician contact to guide them through the process puts first-generation medical students at a distinct disadvantage. Finally, student load debt may be one more factor that adds uncertainty to a first-generation medical student’s experience more so than a non-first-generation medical student. The uncertainty of moving through life with an exorbitant amount of debt can produce a lot of anxiety [42]. Students whose family members are doctors have the means, if they choose, to help students pay for medical school. This option is less likely for first-generation medical students. Not everyone who starts medical school makes it to graduation. For a first-generation medical student, having this accumulating debt as they move through medical school makes the prospect of dismissal or leaving voluntarily all the more distressing. This uncertainty for the future can manifest in negative ways such as generalized anxiety disorder and obsessive-compulsive disorder [43–46]. Thus, identifying medical students with higher intolerance of uncertainty and prospective intolerance of uncertainty is critical to provide a supportive learning environment that promotes psychological safety [47]. More research is needed to examine psychological, academic, financial, and social barriers and their associations with uncertainty.

Finally, we observed differences in grit, self-efficacy, curiosity, and intolerance of uncertainty by age, gender, race, and year in medical school. The purpose of this study was not to examine differences in these factors by demographic variables; however, we felt it necessary to examine these relationships to create our final regression model. While it is important to control for demographic variables, observed differences do not equate true differences by groups. For example, both gender and race are social constructs that are used to categorize people based on physical characteristics and behavioral patterns; thus, observed differences do not represent a biological or genetic differences. Rather, observed differences in grit, self-efficacy, curiosity, and intolerance of uncertainty by gender and race may suggest systemic discrimination. Assessing the complexity of sexism and racism in medical education and their relationship to grit, self-efficacy, curiosity, and intolerance of uncertainty is beyond the scope of this study. Future research should examine these relationships to ensure that medical education serves the needs of all students.

## Limitations

The current study had several limitations, including the cross-sectional study design, homogeneity of the study sample from one osteopathic medical school with three campuses, selection bias, social desirability bias, and a lack of psychosocial and academic performance measures. The cross-sectional study design prevented determinations of causality among first-generation status and grit, general self-efficacy, curiosity and exploration, and intolerance of uncertainty. Data from one osteopathic medical school from a predominantly White sample limits the ability to generalize the findings to other medical schools. Further, the response rate (51.5%) was moderate, and therefore the findings are susceptible to selection bias. Additionally, participants may have responded to questions in a manner that they believed would be viewed more favorably by others, thus creating social desirability bias. In an attempt to minimize social desirability bias, no researchers were present when the participants completed the survey and participation was anonymous. Moreover, longitudinal research should assess these constructs at multiple time points throughout the year with a larger, more racially and ethnically diverse sample of medical students, from multiple schools in different geographic regions. Future research must also account for the effect of the SARS-CoV-2 pandemic on these factors, especially intolerance of uncertainty. Lastly, we did not assess psychosocial outcomes or current academic performance. Including these measures in future research are needed to strengthen the quality of the study as well as provide a more complete picture of how these constructs influence medical student outcomes.

## Conclusions

These findings suggest that first-generation statuses did not predict grit, self-efficacy, curiosity and exploration, or intolerance of uncertainty. Statistical trends were observed with first-generation medical students predicting higher intolerance of uncertainty and prospective intolerance of uncertainty. Additional research with a larger, more diverse sample assessing these constructs, psychosocial outcomes, and academic performance is needed to draw meaningful conclusions.

## Data Availability

The dataset generated and analyzed for the current study are not available for public use; however, they are available upon request from the corresponding author.
